# Super-Resolution Microscopy: Shedding New Light on *In Vivo* Imaging

**DOI:** 10.3389/fchem.2021.746900

**Published:** 2021-09-14

**Authors:** Yingying Jing, Chenshuang Zhang, Bin Yu, Danying Lin, Junle Qu

**Affiliations:** Key Laboratory of Optoelectronic Devices and Systems of Ministry of Education and Guangdong Province, College of Physics and Optoelectronic Engineering, Shenzhen University, Shenzhen, China

**Keywords:** super-resolution techniques, *in vivo* imaging, labeling strategies, near-infrared fluorescent probes, *in vivo* applications

## Abstract

Over the past two decades, super-resolution microscopy (SRM), which offered a significant improvement in resolution over conventional light microscopy, has become a powerful tool to visualize biological activities in both fixed and living cells. However, completely understanding biological processes requires studying cells in a physiological context at high spatiotemporal resolution. Recently, SRM has showcased its ability to observe the detailed structures and dynamics in living species. Here we summarized recent technical advancements in SRM that have been successfully applied to *in vivo* imaging. Then, improvements in the labeling strategies are discussed together with the spectroscopic and chemical demands of the fluorophores. Finally, we broadly reviewed the current applications for super-resolution techniques in living species and highlighted some inherent challenges faced in this emerging field. We hope that this review could serve as an ideal reference for researchers as well as beginners in the relevant field of *in vivo* super resolution imaging.

## Introduction

Cells are the functional units of life and grow in a relative complicated environment. Getting accurate physiological knowledge requires studying cells within their parent organisms, and thus scientific researchers could obtain the biological information in a native state within the organism itself, where all the cues are driven by gene expression (Liu et al., 2018). While many of the structures and organization patterns of biomolecules in *in-vitro* cells have been identified, *in vivo* studies will offer unique insights into the cellular morphology in relation to genetical conditions or pathological modifications (Vacaru et al., 2014; Stone et al., 2017; Zheng et al., 2020). However, at present, it is still a challenge to observe the organelles and macromolecular complexes *in vivo* at high resolution.

So far, fluorescence microscopy has been an unprecedented choice for *in vivo* studies as it offers noninvasive imaging, good specificity and high temporal resolution (Sigrist and Sabatini, 2012). Traditional imaging tools such as confocal microscopy and multiphoton microscopy have been employed to study vital physiological activities in living systems (Heilemann, 2010). Nevertheless, more detailed measurement of morphological changes in living species has been hindered by the diffraction limitation of light, which is about half the wavelength of light (200–300 nm) (Hell et al., 2004; Agrawal et al., 2013). Hence, sub-diffraction imaging techniques are needed urgently for investigating the fine structures *in vivo*.

Over the past 2 decades, advances in super-resolution microscopy (SRM) have revolutionized the field of fluorescent imaging and become valuable tools in biological studies (Coltharp and Xiao, 2012; Baddeley and Bewersdorf, 2018; Schermelleh et al., 2019). These SRM techniques are most prominently harnessed in two distinct families: one employs patterned illumination to spatially modulate the fluorescence behavior and their related derivatives, such as stimulated emission depletion microscopy (STED) (Hell and Wichmann, 1994; Vicidomini et al., 2018), structured illumination microscopy (SIM) (Gustafsson, 2000; Li et al., 2015; Turcotte et al., 2019) and reversible saturable optically linear fluorescence transitions (RESOLFTs) (Grotjohann et al., 2011); the other obtains super-resolution images based on the localization of individual emitting molecules, such as stochastic optical reconstruction microscopy (STORM) (Rust et al., 2006; Bates et al., 2013) and photoactivated localization microscopy (PALM) (Betzig et al., 2006; Henriques et al., 2011). Now, SRM systems have become central tools in the biomedical research community (Coltharp and Xiao, 2012; Muller and Heilemann, 2013; Stone et al., 2017; Xu J. et al., 2020).

Despite significant progress, the application of present SRM techniques to scattering tissues or living samples are limited due to poor imaging depth. The unnecessary excitation of out-of-focus fluorophores degrades the quality of the in-focus signal, making it difficult to resolve the fine structures. Besides, the imaging rates are still insufficient for many *in vivo* applications, especially those involving signal transportation studies (Galbraith and Galbraith, 2011; Biteen and Willets, 2017). Moreover, even in the absence of aberrations, most SRMs achieve high resolution usually by intense illumination, which can disturb delicate subcellular processes or even introduce permanent phototoxic damages (Hell et al., 2015; Kilian et al., 2018). Consequently, the capabilities of SRM implementations are hampered in the field of *in vivo* imaging.

Even though, to address these challenges, considerable efforts have been extended toward enhancing the imaging depth and developing highly sensitive fluorescent probes to realize real-time imaging *in vivo*. For example, Adaptive Optics (AO) has been introduced in many SRM techniques to enhance the imaging depth by eliminating the sample-induced distortions in the wavefront using a dynamically reconfigurable optical element. Nowadays, the improvements in optical system construction, camera technologies and labeling methods have made SRM possible to visualize physiological activity in living organism. In the following pages, we provide an overview of recent advances in SRM techniques for imaging *in vivo*. The technological modifications in the burgeoning field of “*in vivo* SRM” are first clarified, and the current fluorescent probes for *in vivo* SRM labeling are described. Then, the applications of these techniques in various biological areas are discussed, and the challenges as well as future trends in this emerging field are highlighted.

## Super-Resolution Techniques for *In Vivo* Imaging

### Stimulated Emission Depletion (STED) Microscopy

**FIGURE 1:**
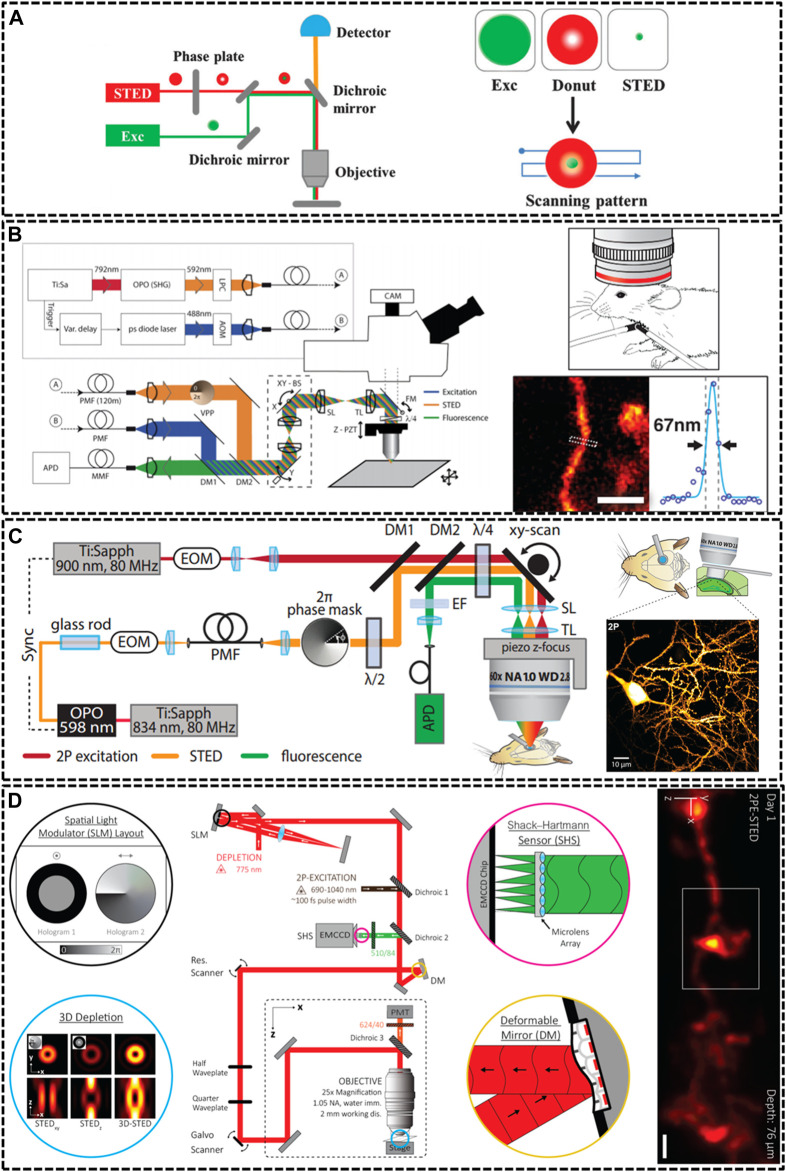
Overview of modified STED microscopy for *in vivo* imaging. **(A)** The principle of STED technique. The combination of excitation laser and donut-shaped STED laser could decrease the size of PSF effectively (Yang et al., 2016). Reprinted from Yang et al. (2016) with permission from Royal Society of Chemistry. **(B)** Schematic drawing of the upright scanning STED for *in vivo* imaging (Berning et al., 2012). Reprinted from Berning et al. (2012) with permission from American Association for the Advancement of Science. The STED image of dendritic and axonal structures was obtained from a living mouse brain under the objective lens (1.3 NA, glycerol immersion). Scale bar, 1 μm. **(C)** Diagram of two photon (2P) STED microscope (Pfeiffer et al., 2018). The 2P image depicted dendrites of pyramidal neuron in hippocampal CA1 region. Scale bar, 10 μm. Reprinted from Pfeiffer et al. (2018) with permission from eLife Sciences. **(D)** Schematic of aberration-corrected 3D-2P-STED instrument (Velasco et al., 2021). The microscopy equipped with a double-pass spatial light modulator (SLM) to impart both the vortex and top-hat phase masks on the depletion beam. Super-resolution image showed a 3D dendrite at 76 µm below the cortical surface. Scale bar: 1 µm. Reprinted from Velasco et al. (2021) with permission from Optical Society of America.

In 1994, the concept of STED microscopy was first proposed and it was subsequently demonstrated experimentally (Hell and Wichmann, 1994). Briefly, STED microscopy is a two-beam technique that applies a STED beam to suppress the emission of the fluorescent molecules located off the core of the excitation region in order to sharpen the effective point spread function (PSF) (Figure 1A) (Yang et al., 2016). On that account, the depletion beam needs an illumination pattern shaped like doughnut with non-zero intensity at the periphery and zero intensity at the center of the excitation spot. Scanning the sharpened PSFs across the sample could allow recording a super-resolved image with high resolution. As an extended technique based on confocal microscopy, STED could obtain the images directly, and thus is better suited for *in vivo* imaging (Jahr et al., 2020; Steffens et al., 2020). Moreover, the inherent 3D sectioning capability of STED makes it ideal for whole tissue imaging (Hell and Wichmann, 1994; Spahn et al., 2019).

Because of specimen-induced scattering distortion and aberrations, there is a great challenge for STED microscopy to maintain consistent resolution in deep tissue inside living bodies. In 2011, Stefan W. Hell’s group modified STED microscopy equipping with a high-numerical aperture (NA) glycerol immersion objective lens and a correction collar to improve the penetration depth. They could image the actin at a depth of 120 μm below the tissue surface inside living brain slices with a spatial resolution of 60–80 nm (Urban et al., 2011). Afterwards, the real *in vivo* STED microscopy was first demonstrated also by his group in 2012 (Berning et al., 2012). They developed an upright scanning STED microscope with a 1.3 NA objective lens focusing 488 nm wavelength in a mouse brain (Figure 1B). The focused excitation pulses were aligned and synchronized with the doughnut-shaped 592 nm STED pulses for silencing the enhanced yellow fluorescent protein (EYFP). This STED technique was successfully applied to image the 10–15 μm molecular layer below the surface with a resolution of about 70 nm. Later, a few groups also modified STED technique to increase the imaging depth through various methods. For example, Kebin Shi’s group reported a Gaussian-Bessel STED (GB-STED) microscopy, which achieved an imaging depth of 100 μm in brain tissue by modulating the depletion beam into a hollow Bessel beam and using a conventional Gaussian beam for excitation (Yu et al., 2016).

Two-photon (2P) excitation is important for *in vivo* imaging as it is well suited for deep tissue observation (Helmchen and Denk, 2005). The main strengths of 2P excitation include that typical 2P laser scanning microscopy could increase imaging depth by using near infrared (NIR) excitation laser beam, and the 2P excitation could reduce background by confining excitation to the focal point of the objective (Denk et al., 1990; So et al., 2000). Thus, U Valentin Nagerl et al. combined 2P excitation with STED (2P-STED) microscopy to improve the imaging depth and resolution, exploiting new perspectives for deep tissue imaging both *in vitro* and *in vivo* (Bethge et al., 2013; Ter Veer et al., 2017). In 2013, they constructed 2P-STED microscope incorporating with a pulsed STED laser, a long-working distance water objective and spectral detection for two-color imaging in brain slices (Bethge et al., 2013). The symmetry and central minimum of the STED doughnut were optimized by adjusting a λ/4 wave plate in front of the scanner. Then, in 2018, they successfully applied the home-built 2P-STED microscope to reach the deeply located hippocampus *in vivo* (Figure 1C) (Pfeiffer et al., 2018).

Most life live by means of three-dimensional interplay with millions of components, and thus defining their structural details not only requires subdiffraction resolution in x-y plane, but also along demands high optical resolution in z axis (Lin et al., 2018). Unfortunately, most current depletion effects of PSF remains diffraction limited in the z direction. Several methods have been implemented to improve the axial resolution of STED microscopy, such as 4Pi-STED, isoSTED and the method using the top-hat phase mask (Huang et al., 2010; Galbraith and Galbraith, 2011; Vicidomini et al., 2018). However, these methods still limited the applications of STED in deep-tissue imaging in living specimens. In 2019, Jason R. Swedlow et al. demonstrated a STED microscope that was capable of 3D super-resolution imaging with automated aberration correction (Zdankowski et al., 2020). They introduced an image denoising method based on block-matching and collaborative 3D filtering (BM3D) to numerically enhance fine object details. The technique achieved lateral and axial resolution of 204 and 310 nm in an 80 μm thick layer of tissue. As noted above, AO could enhance imaging depth by adaptively correcting aberration induced from the sample, therefore, it plays a vital role in in vivo imaging. Recent work impressively demonstrated AO-improved STED microscopy of aberrating samples. Joerg Bewersdorf et al. have achieved aberration-corrected 3D STED imaging at 76 µm depth through the combination of 2P excitation, AO correction, organic dye labeling approach, and a long-working-distance water-immersion objective lens (Velasco et al., 2021). As shown in Figure 1D, they employed an AO architecture based on wavefront sensing to correct aberrations. Moreover, the 2-mm working distance of objective lens accommodated a wide range of sample dimensions and configurations and allowed long penetration depths into the living species. For 2P excitation, they used light from a femtosecond (fs)-pulsed titanium sapphire laser that was fused with the STED beam via a dichroic mirror. The 3D-2P-STED was successfully applied to the brain of a living mouse with an axial resolution of 321 nm.

### Reversible Saturable Optical Fluorescence Transition

**FIGURE 2:**
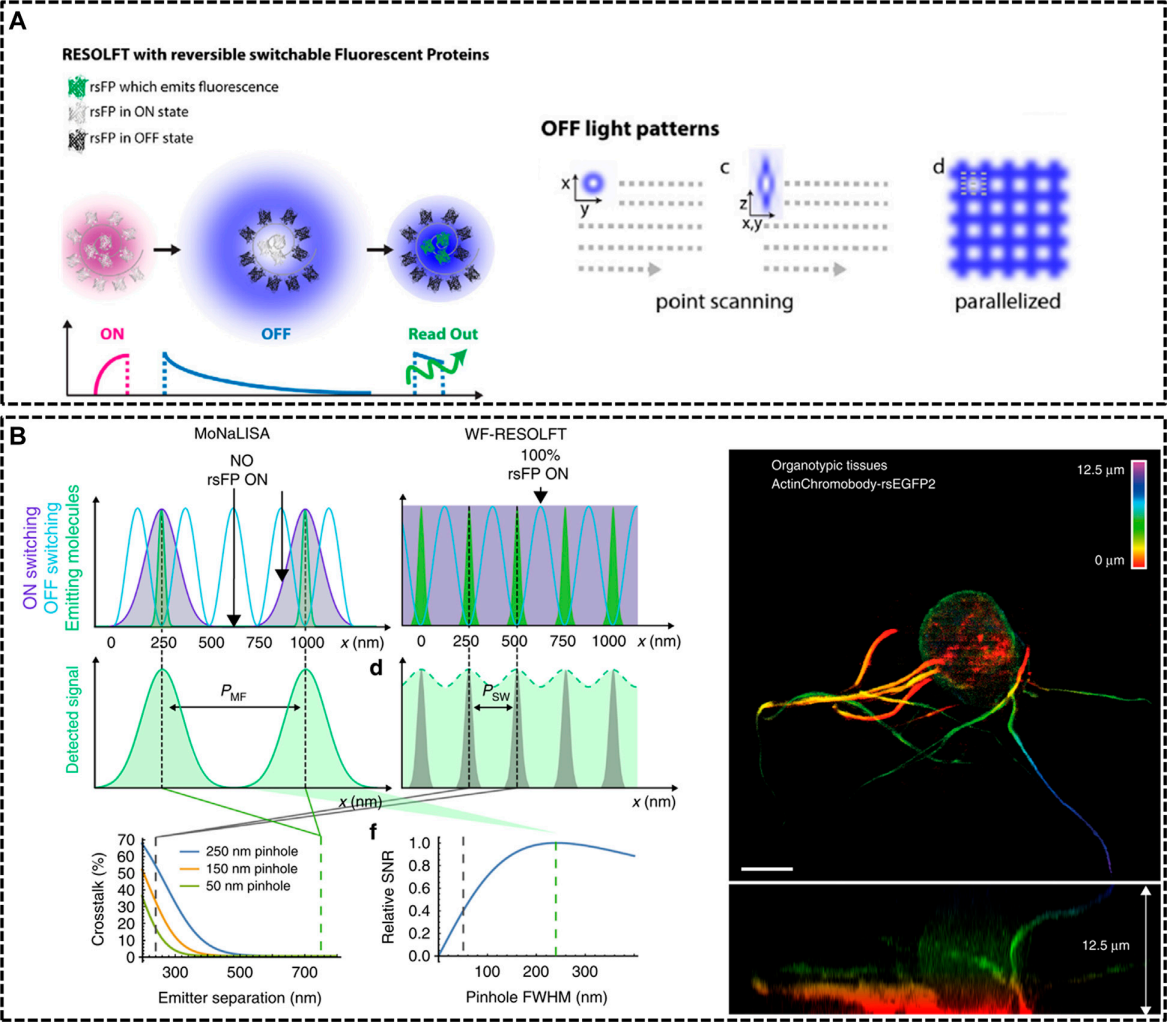
Modified RESOLFT techniques with reversible photoswitchable fluorescent proteins for *in vivo* imaging. **(A)** Left: illustration of the typical pulse mode for RESOLFT. Right: Off light pattern implemented in point scanning and parallelized RESOLFT microscopy (Hell et al., 2015). Reprinted from Hell et al. (2015) with permission from IOP Publishing. **(B)** Schematic representation of Molecular Nanoscale Live Imaging with Sectioning Ability (MoNaLISA) and the actin recorded in a 12.5 μm organotypic hippocampal rodent brain tissue (Masullo et al., 2018). Scale bar, 5 μm. Reprinted from Masullo et al. (2018) with permission from Springer Nature.

The depletion concept has been extended to photoswitchable fluorescent proteins by using the donut-shaped depletion beam to switch fluorophores into the OFF state instead of stimulated emission, which is known as RESOLFT microscopy (Grotjohann et al., 2011; Sharma et al., 2020). The RESOLFT technique employs the genetically encoded markers and minimizes the illumination light intensities, thus it is suitable for discerning molecular structures and individual organelles in the interior of living body. The spatial resolution could be indeed improved through saturating the OFF-switching transition by using a light pattern featuring one or more intensity minima or “zeros” (Figure 2A) (Hell et al., 2015). Unlike STED which requires an intense depletion laser, a low saturation intensity was chosen for RESOLFT, permitting much lower phototoxic damage (Grotjohann et al., 2011).

In 2012, Stefan W. Hell’s group constructed a RESOLFT setup equipping with dichroic mirrors and filters dedicated for the green fluorescent protein Dronpa-M159T (Testa et al., 2012). The reversibly switchable fluorescent protein (rsFP) Dronpa-M159T was switched on with 405 nm wavelength and switched off by a counterpart of 491 nm wavelength featuring a doughnut shape with central zero intensity, and excited with another light, also of 491 nm illumination. Dendritic spines were recorded for hours inside living organotypic hippocampal brain slices. Moreover, in 2018, Ilaria Testa et al. developed Molecular Nanoscale Live Imaging with Sectioning Ability (MoNaLISA) to image structures in organotypic tissues (Masullo et al., 2018). Principle of MoNaLISA was based on RESOFT but had a little difference. The MoNaLISA imaging employed three light illuminations for ON-switching, OFF-switching, and read-out of the rsFPs, respectively. The ON-switching and read-out pattern contained multiple individual foci which are divided by the same multi-foci periodicity (P_MF_). The OFF-switching mode, which is responsible for sharpening the PSF with multiple intensity minima, features standing waves at a periodicity of P_SW_. The independent light patterns could adjust the light doses of the OFF pattern by choosing the smallest P_SW_ and select the optimum P_MF_ to enhance the photon collection and the sectioning. By maximizing the detected photon flux, MoNaLISA enabled long-time and large field-of-view (50 × 50 μm^2^) recordings at 15 µm depth with a resolution of 70–100 nm (Figure 2B).

### Structured Illumination Microscopy

**FIGURE 3:**
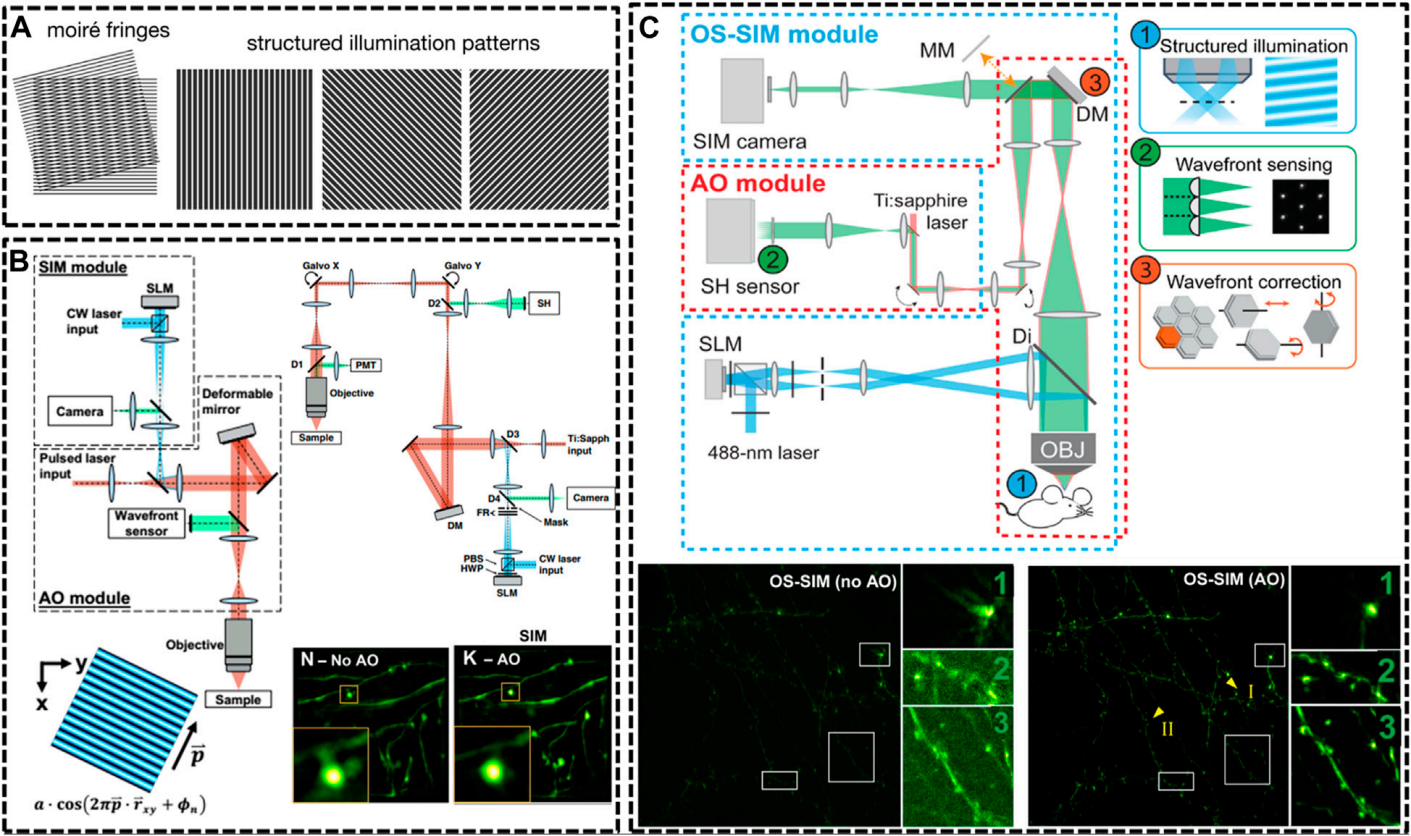
Schematic of improved setups of SIM for *in vivo* imaging. **(A)** Illustration of SIM to resolve an unknown structure with a known periodical pattern (Sezgin, 2017). Reprinted from Sezgin (2017) with permission from IOP Publishing. **(B)** Detailed optical layout of the SIM setup and minimization of aberrations via correction collar and adaptive optics (AO) (Turcotte et al., 2019) Reprinted from Turcotte et al. (2019) with permission from National Academy of Sciences of the United States of America. **(C)** An AO module applied to optical-sectioning structured illumination microscopy (OS-SIM), which enables a marked improvement in resolution, signal, and contrast for imaging spine in mouse brain (Li et al., 2020). Scale bar, 10 μm in original images; 3 μm in magnified box region. Reprinted from Li et al. (2020) with permission from American Association for the Advancement of Science.

SIM extracts fine structural details from the interference of a structure with predesigned illumination patterns (Gustafsson, 2000; Galbraith and Galbraith, 2011; Huang et al., 2009). This light pattern could rotate into different angles and move along the sample. A super-resolved SIM image with high spatial resolution could be reconstructed from a series of frames with appropriate algorithms (Figure 3A) (Sezgin, 2017; Heilemann, 2010). Although the resolution is limited to ∼100 nm, SIM has wide applications as it does not need special labelling molecules and can work with common fluorophores unlike other SRM techniques (i.e., fairly photostable probes for STED and photoswitchable probes for PALM/STORM) (Fernandez-Suarez and Ting, 2008; Brunstein et al., 2013; Dan et al., 2014).

In 2012, Hari shroff’s group developed a multifocal SIM (MSIM) for super resolution imaging at depths of greater than 45 μm in live transgenic zebrafish embryos (York et al., 2012). They chose a commercially available digital micromirror device (DMD) to generate and switch multifocal patterns. MSIM data acquisition and processing were conceptually divided into multiple steps: 1) exciting the sample with a sparse and multifocal illumination pattern; 2) employing digital pinholes around each fluorescent focus; 3) 2 x scaling; 4) repeating above steps until whole sample has been fully excited; and 5) accumulating all such pinholed and scaled images. Following this outstanding work, they continued to generate an instant SIM technique that permitted acquisition and display of super-resolution images in real time (York et al., 2013). The key concept of realizing instant SIM is that every step of digital combination in MSIM is performed optically. They then applied the instant SIM visualize cytoskeletal detail within flowing blood cells *in vivo* at an unpresented speed. As mentioned above, 2P excitation is an effective way to reduce the effect of optical scattering, causing deeper imaging depth into tissues when compared to single-photon illumination. In 2018, Zhang et al. developed the resonant two-photon Super resolution Patterned Excitation Reconstruction (2P-SuPER) microscopy to realize the observation of the dendrite at depth of up to 120 μm below the brain surface with an imaging rate of 3.5 Hz (Urban et al., 2018).

Afterwards, a few new strategies have been implemented in SIM technique for *in vivo* imaging. In 2019, Na Ji’s group developed AO corrected SIM to image the brains of live zebrafish larvae and mice at nanoscale resolution (Turcotte et al., 2019). The optical system for *in vivo* SIM imaging consisted of two modules: one for SIM itself and one for AO to compensating specimen-induced aberrations. With these optimizations, they were able to routinely image sparsely labeled neural structures at a depth of 50 µm (Figure 3B). Then, in the next year, they further optimized the optical-sectioning SIM (OS-SIM) technique for imaging *in vivo* through modifying the reconstruction algorithms, correcting motion-induced artifacts and incorporating an AO module (Li et al., 2020). With AO OS-SIM, they demonstrated fast, high-resolution imaging for structural details of mouse cortical neurons *in vivo* (Figure 3C).

### Lattice Light Sheet Microscopy

**FIGURE 4:**
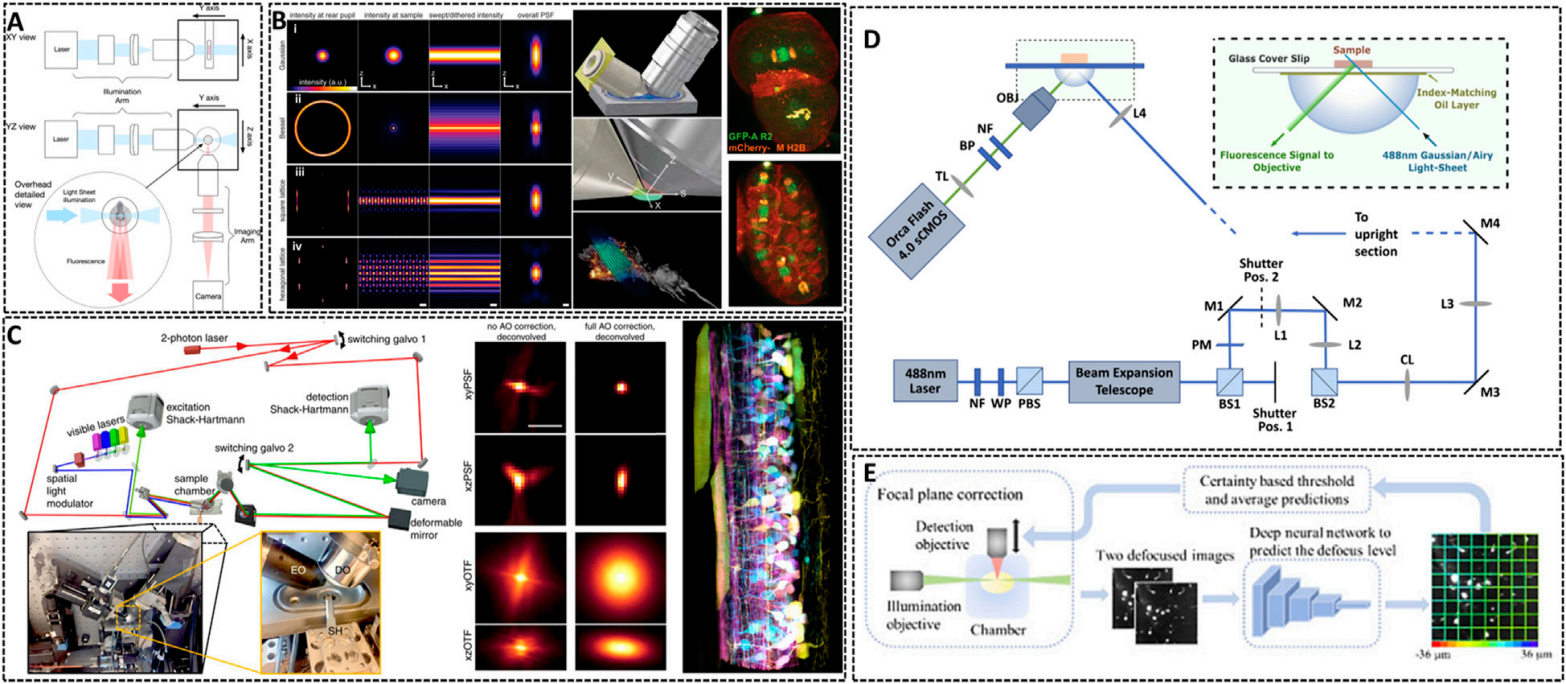
Lattice light-sheet microscopy for *in vivo* imaging. **(A)** Basic working principle for light-sheet microscopy method (Girkin and Carvalho, 2018). The excitation light sheet is generated using a cylindrical lens before the excitation objective and the orthogonal imaging arm. Reprinted from Girkin and Carvalho, (2018) with permission from IOP Publishing. **(B)** Left: methods showing the traditional approach (Gaussian beam, i), Bessel beam (ii), and square (iii) and hexagonal lattice (iv) used for light sheet microscopy (Chen B.-C. et al., 2014). Middle: scheme illustrating the core of light sheet microscopy. Right: distribution of chromosomal passenger protein GFP-AIR-2 (green) relative to plasma membranes and histones (red) in C. elegans embryos at the two-cell stage and the six-cell stage. Reprinted from Chen et al. (2014) with permission from American Association for the Advancement of Science. **(C)** Left: simplified microscope schematic of adaptive optical lattice light-sheet microscopy (AO-LLSM) (Liu et al., 2018). Midddle: maximum intensity projections (MIPs) and corresponding optical transfer function (OTFs) of the uncorrected and fully corrected bead images. Scale bar, 1 μm. Right: image of the spinal cord of a zebrafish embryo 58 hpf. Reprinted from Liu et al. (2018) with permission from American Association for the Advancement of Science. **(D)** Schematic of the setup of LSM using an Airy beam (Corsetti et al., 2020). Reprinted from Corsetti et al. (2020) with permission from Optical Society of America. **(E)** Overview of the integration of the deep learning-based autofocus method with a custom-built LSM (Li et al., 2021). Reprinted from Li et al. (2021) with permission from Optical Society of America.

Light-Sheet Microscopy (LSM) is a vital technology that is suitable for observing living tissue or whole organism with the advantages of deep penetration depth, high contrast, low phototoxicity, and fast acquisition rate (Adams et al., 2015). LSM realizes high-resolution images by confining a sheet of light within the specimen, which coincides with the focal plane of a high NA objective placed at 90 (Figure 4A) (Girkin and Carvalho, 2018; Albert-Smet et al., 2019; Manley et al., 2020). Now, LSM has been successfully applied to record the early stages of fly, mouse embryos and activities in the entire spinal cord of live zebrafish embryos *in vivo* (Chen B.-C. et al., 2014; Liu et al., 2018). More recently, light sheet-based imaging patterns have successfully been coupled with super-resolution imaging paradigms which allow imaging beyond the diffraction limit (Gustavsson et al., 2018; Wang et al., 2021).

The way of generating this light sheet will determine the sensitive degree of the microscopy to scattering (Gao et al., 2019). Conventional light sheets created with Gaussian beams are too thick over cellular scale to enable subcellular observation. In 2014, Eric Betzig’s group developed Lattice Light Sheet Microscopy (LLSM) by using 2D optical lattices, which allows for four-dimensional (4D) (x, y, z, and time) imaging with exceptionally high temporal resolution (∼100 frames/s, ∼1 cell volume/s) and minimal photobleaching (Chen B.-C. et al., 2014). As shown in Figure 4B, LLSM utilized an isolated excitation lens which was perpendicular to the widefield detection lens to confine the illumination to the neighboring focal plane. The lattice light sheet intersects the specimen obliquely for the sake of applying such light sheets in *in vivo* imaging. LLSM works in two modes: one is a dithered mode, in which an optical lattice is scanned continuously for high-speed 3D imaging; the other is a super-resolution structured illumination microscopy (SR-SIM) mode, in which an optical lattice is scanned discretely to enhance the spatial resolution. The SR-SIM mode provided a high spatiotemporal resolution for imaging embryogenesis in *Caenorhabditis elegans* at the two-cell stage and the six-cell stage (Figure 4B, right). Further, the same group continued to introduce an independent AO module in their LLSM system to decrease the aberrations of light (Liu et al., 2018). The sample-induced aberrations were first measured by creating a reference “guide star” through two-photon excited fluorescence (TPEF), and then corrected with a phase modulation element. Coupling with correction times as short as 70 ms, this AO module is compatible with the speed and noninvasiveness properties of LLSM. One of the main advantages of complete AO correction is that it enables accurate deconvolution (Figure 4C), ensuring the most truthful representation of the species within the diffraction limits. With 3D AO-LLSM, they successfully visualize a living zebrafish embryo across 200 μm depth.

Although the unique and orthogonal excitation-detection pattern of LSM makes it fast to record images with a deep imaging depth *in vivo*, there are some limitations for LSM to acquire high-quality images, such as the restrained image resolution, spatial heterogeneity in the refractive index of the specimen and artifact-prone reconstruction process for time-lapse imaging. Remarkable, deep learning approach has become a promising method to resolve above problems and is applied in LSM to enhance the resolution and discover intricate structures (Wagner et al., 2021). Dholakia’s group combined the use of a non-diffracting Airy light field and a deep-learning method to demonstrate an open-top light sheet microscopy (Figure 4D) (Corsetti et al., 2020). The adoption of deep-learning in their system has demonstrated a near two-fold improvement in resolution whilst maintaining a wide field of view for light sheet imaging. Li et al. introduced a deep learning-based autofocus framework that can estimate the position of the objective-lens focal plane relative to the light-sheet (Figure 4E) (Li et al., 2021). They realized a large 3D specimens imaging with high spatial resolution.

### Ultrasound Localization Microscopy

**FIGURE 5:**
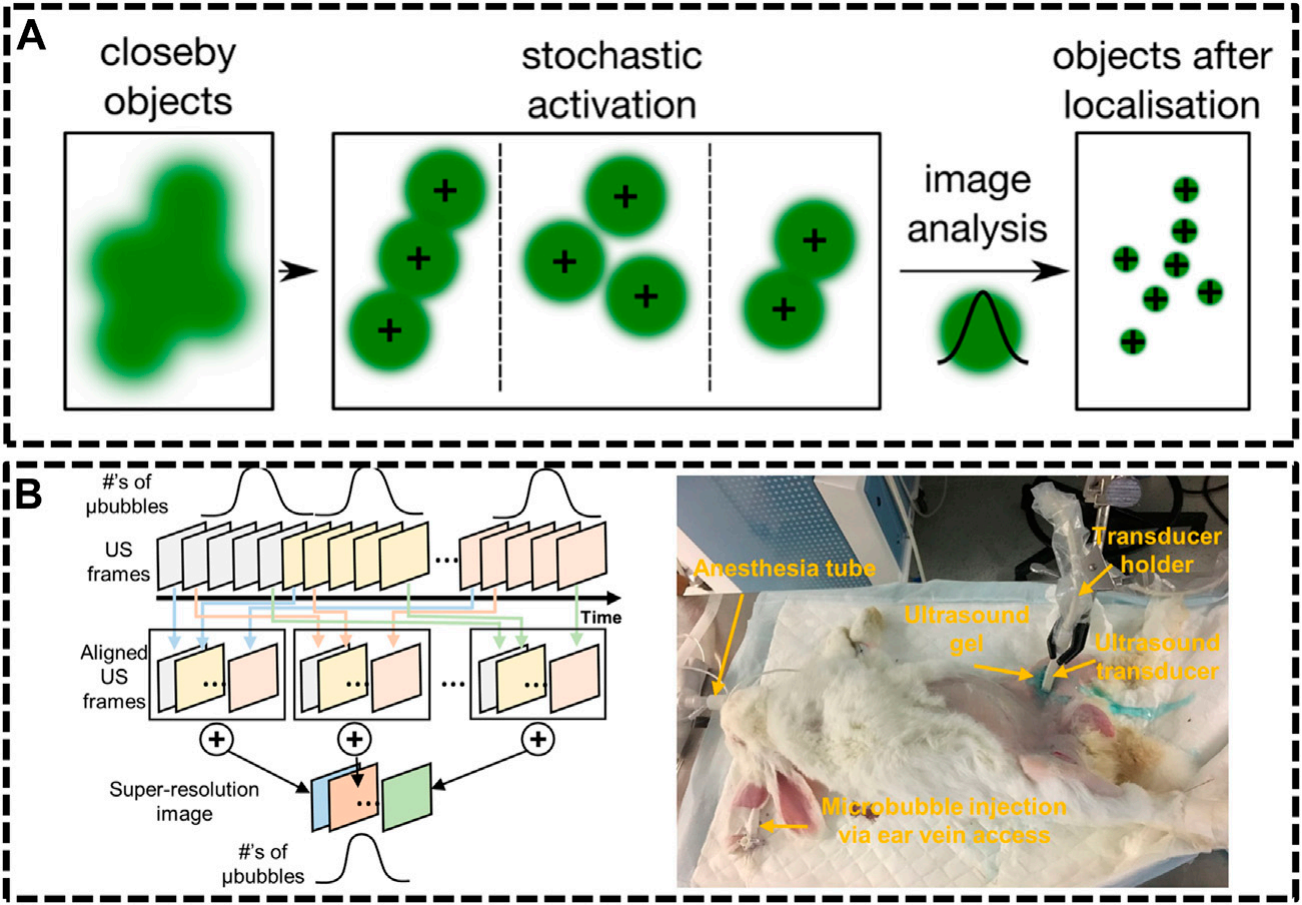
Ultrasound localization microscopy (ULM) systems. **(A)** Principle of single molecule localization microscopies (SMLMs) (Sezgin, 2017). Reprinted from Sezgin (2017) with permission from IOP Publishing. **(B)** Graphical diagram of super-resolution ultrasound data acquisition and experiment setup of rabbit imaging (Yu et al., 2018). Reprinted from Yu et al. (2018) with permission from Springer Nature.

In conventional fluorescence microscopes, most illuminated probes emit fluorescence at the same time, resulting an overlay of PSF of several individual fluorophores. Switching on only a random and small subpopulation of fluorescent molecules in each frame could ideally decrease the density of emitting signals, and thus, the neighboring fluorescent emitters do not overlap and each indivi dual PSF can be isolated and determined with subdiffraction accuracy (Figure 5A) (Sezgin, 2017). Single molecule localization microscopies (SMLMs), including PALM and STORM, carry out the above principle and employ photoswitchable fluorophores to generate super-resolved images (Sahl and Moerner, 2013; Sauer, 2013). However, SMLMs acquire thousands of frames to reconstruct a single plane, and the associated long acquisition time, as well as the limited photoswitchable probes restrict the general applicability of SMLMs for live-cell or *in vivo* imaging.

Compared with optics, ultrasonic waves could spread deep into tissues maintaining their coherence and are much less influenced by the irrelevant processes *in vivo*. Nevertheless, the resolution of ultrasound imaging technique is impeded by diffraction limitation, which endures a long-standing trade-off between resolution and imaging depth. Ultrasound Super Resolution Microscopy (USRM) has been recently introduced to overcome the diffraction limitation defined by the acoustic limit (Yoon et al., 2018; Yu et al., 2018). This approach combined the single localization principle with ultrasound technologies. For example, microbubbles were injected to tissue and each microbubble as shown in could be considered as a point source (Errico et al., 2015). The recorded echo signal from individual microbubbles is representation of the PSFs of the points. A super-resolved image can be reconstructed by accumulating the PSF of each separable source over thousands of frames. In 2015, Mickael Tanter et al. demonstrated an ultrafast Ultrasound Localization Microscopy (ULM) to visualize the microvessels *in vivo* by gas microbubbles inserting technology (Errico et al., 2015). The bubbles created separated and rapid-changing localizations within the ultrasound images, which were detected using frame-to-frame differential processing. Each entire ultrasonic frame was generated by using parallel beamforming. As these bubbles are uniquely located, enough positions were recorded to reconstruct a super resolution image through a thinned skull at a coronal section, providing a resolution of 10 μm in depth and 8 μm in lateral direction. Then, in Yu et al. (2018) developed an imaging sequence and signal processing approach to enhance temporal resolution by applying deconvolution and spatio-temporal-interframe-correlation (STIC) based data acquisition (Yu et al., 2018). The STIC method was used to compensate motion over reduced data acquisition time. This technique was demonstrated in the rabbit atherosclerosis model (Figure 5B).

Several groups subsequently proposed advanced strategies to improve the frame rate. Xuejun Qian’s group developed ultrafast plane wave imaging approach for mapping vasculature using image deconvolution method (Qian et al., 2020). The imaging setup equipped with a high frequency Verasonics Vantage system and a linear array transducer. In order to adjust the distance to imaging targets, the array transducer was mounted on a 3-axis translation motorized linear stage system with a minimum step size of 60 nm. 2D/3D-view images of rabbit eyes *in vivo* were performed to demonstrate the capability of this technique, providing the vasculature network of the posterior pole of the eye, especially for choroidal and retrobulbar vessels.

## Labeling Approaches

### Genetically Encoded Probes

The main bottleneck to apply super-resolution techniques *in vivo* is seeking for appropriate fluorescent probes with deep tissue penetration, high brightness and photostability. Nowadays, many types of fluorophores have been developed for SRM, including fluorescent proteins (FPs), dyes and nanoparticles (Heilemann et al., 2008; Shcherbakova et al., 2014; Wang et al., 2018). However, stringent requirements are needed for their use in SRMs *in vivo*. Much effort has been devoted to the development of advanced labeling protocols, including adeno-associated virus (AAV) vectors, transgenic mice, and inorganic dyes. In the following, we will highlight our discussion to those labeling approaches applicable for *in vivo* SRM.

**FIGURE 6:**
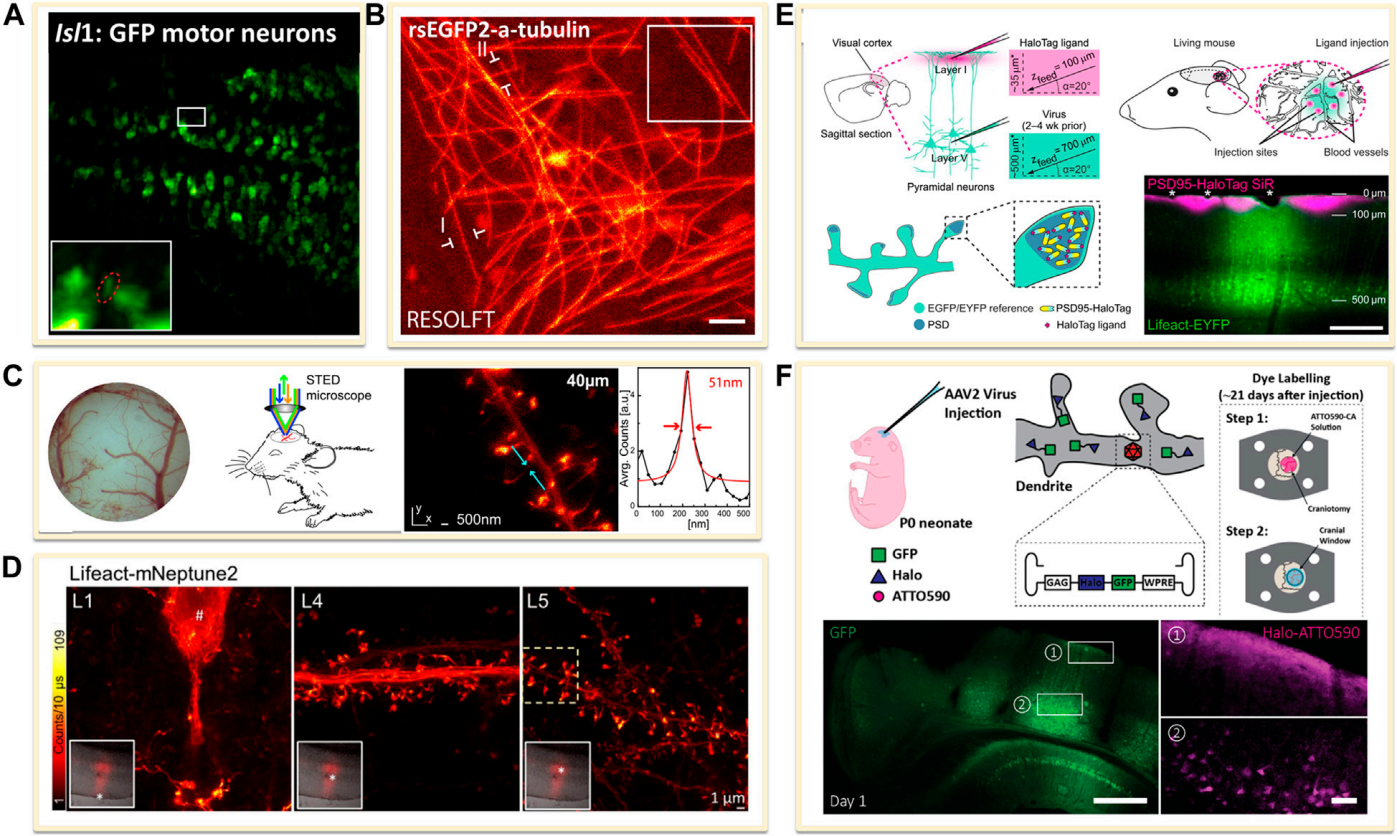
Genetically encoded approaches for *in vivo* labeling. **(A)** Transfected dense Isl1:GFP zebrafish motor neurons (Li et al., 2020). Scale bar, 20 μm. Reprinted from Li et al. (2020) with permission from American Association for the Advancement of Science. **(B)** *Drosophila melanogaster* ubiquitously expressing rsEGFP2-a-tubulin (Schnorrenberg et al., 2016). Scale bar, 1 μm. Reprinted from Schnorrenberg et al. (2016) with permission from eLife Sciences. **(C)** Neurons expressing Lifeact-EYFP by recombinant adeno-associated virus (AAV) infection for STED microscopy (Willig et al., 2014). Reprinted from Willig et al. (2014) with permission from Cell Press. **(D)** A far red-emitting fluorescent protein mNeptune2 to expressed with lifeact to label actin filaments (Wegner et al., 2017). Reprinted from Wegner et al. (2017) with permission from Springer Nature. **(E)** *In vivo* labeling process of endogenous PSD95-HaloTag with organic fluorophores SiR (Masch et al., 2018). SiR-Halo was injected to transgenic PSD95-HaloTag mice for obtaining a final imaging depth of ∼35 μm below the cortical surface (magenta). Scale bar: 250 μm. Reprinted from Masch et al. (2018) with permission from National Academy of Sciences of the United States of America. **(F)** Neuron labelling strategy in a living mouse using ATTO590 (Velasco et al., 2021). Scale bars: 500 µm in GFP image and 50 µm in right ATTO590 image. Reprinted from Velasco et al. (2021) with permission from Optical Society of America.

The transgenic technology has revolutionized the studies inside living systems. Up to date, the majority of *in vivo* SRM experiments have been performed with FPs. For using FPs in *in vivo* imaging, there are two major approaches: one is using transgenic animals or directly transferring FPs in animals for imaging *in vivo*. For example, several experiments for *in vivo* SRM applied the heterozygous TgN (Thy1-EYFP) mice expressing enhanced yellow fluorescent protein (EYFP) or GFP, EGFP, which is under the control of the regulatory element from the *thy1* gene (Figure 1A) (Berning et al., 2012; Bethge et al., 2013). Similarly, zebrafish and *Drosophila melanogaster* are the commonly used animals for *in vivo* studies, such as the *Isl1*: GFP zebrafish larvae, in which *Islet-1* promoter enabled the expression of GFP in all postmitotic motor neurons (Figure 6A) (Li et al., 2020), and the rsEGFP2 transfected *Drosophila melanogaster*, expressing rsEGFP2 a-tubulin through a standard phiC31 integrase based germ line transformation procedure (Figure 6B) (Schnorrenberg et al., 2016).

The other is to transfer the FPs into animal body to induce the expression of fused FPs or tags through recombinant adeno-associated virus (rAAV, serotype 2) infection method. For example, to label the filamentous actin in neurons, Lifeact, an actin-binding peptide derived from yeast, was usually used as fusion protein by combining diverse FPs (Riedl et al., 2008). The rAAV of serotype 2, facilitated by the neuron specific human synapsin promoter hSYN and Semliki Forest viruses (SFV), could be created to directly express Lifeact-EYFP into the visual cortex of the mouse. A few weeks after infection, the animals could be imaged under SRM systems (Figure 6C) (Willig et al., 2014). The improvement of the penetration depth and the compatibility of the light in the tissue could be optimized through the use of far red-shifted fluorescent proteins. Katrin I. Willig and co-workers applied a far-red emitting FP mNeptune2 for STED imaging of F-actin filament in the cortex in layer 5 (L5) and up to L1 of a living mouse (Figure 6D) (Wegner et al., 2017).

However, FPs generally have poorer photophysical properties than organic dyes. Thus, it is a promising method to implement the far-red or NIR organic dyes for *in vivo* imaging. Several groups have achieved this goal by the combination of genetical coded method with organic dyes. Prior labeling with dyes, the protein of interest is genetically fused with an engineered enzyme tag such as SNAP-, Halo-, or Clip-tag (Chin, 2014). Then these tags could covalently react with the corresponding fluorescent substrate. For expressing these tags in living body, the use of rAAVs offers flexibility in the labelling scheme. Stefan W. Hell and co-workers introduced the NIR SiR-Halo ligand for specific labeling of PSD95 *in vivo* and obtained super-resolution images with superior signal-to-noise ratio and photostability (Figure 6E) (Masch et al., 2018). Similarly, Joerg Bewersdorf et al. utilized the reaction between Halo Tags with Halo reactive ATTO590-chloroalkane (ATTO590-CA) to label neurons as deep as 174 µm below the cortical surface with excellent signal-to-noise ratio by a 3D-2P-STED system (Figure 6F) (Velasco et al., 2021). With the improvements in organic dyes, the use of organic dyes for *in vivo* SRMs will become more feasible. However, improving probes for future SRM studies in the living animal is still going on.

### Near-Infrared Fluorescent Probes

The ideal probe for live cell imaging should have excellent photophysics and chemical properties, such as high specificity, exquisite photostability and possesses good cell permeability. When used in in vivo, the requirements for florescent probes are even stricter. For the past few decades, super-resolution fluorescence imaging has mainly located in the visible and several used near-infrared I light range (Jin et al., 2018). Compared with the visible, red-shifting fluorophores including NIR I (650–950 nm) and NIR-II (1,000–1700 nm) light, would bear several virtues: 1) far-red light has lower scattering across tissues, enhancing tissue penetration and imaging depth; 2) auto-fluorescence caused by the excitation of molecules, like flavins or haemoglobin, is usually reduced in red-shifted wavelengths; 3) absorption of far-red light is less by the tissue when compared to shorter excitations, causing less phototoxic impact (Lei and Zhang, 2020; Reja et al., 2021). Thus, shifting the excitation and emission wavelength of the probe to the red spectral region is necessary for *in vivo* imaging.

**FIGURE 7:**
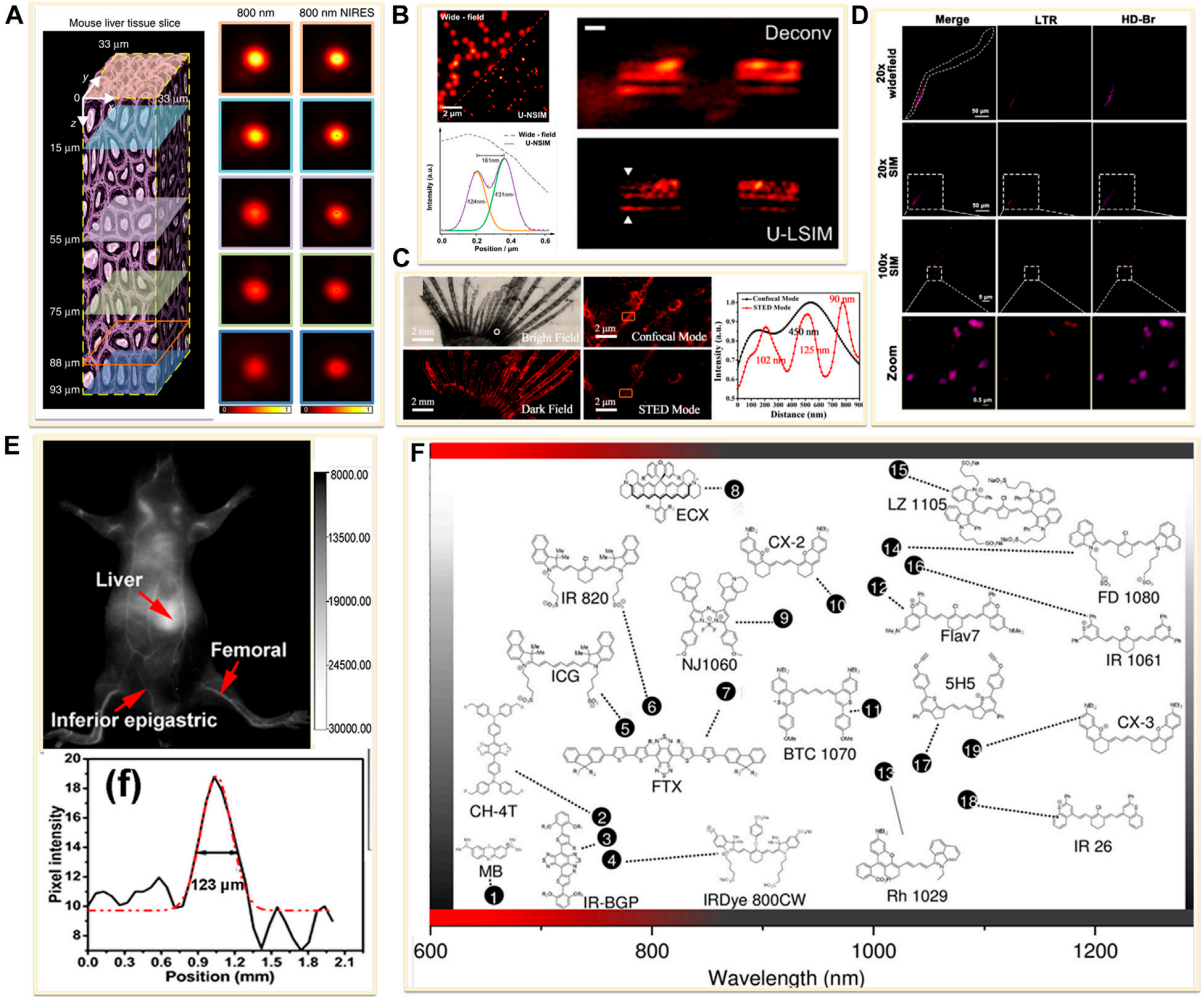
Examples of near-infrared fluorescent probes for *in vivo* imaging. **(A)** Left: diagram of a mouse liver tissue slice with 93 µm thickness (Chen et al., 2018). Right: confocal images and the corresponding near-infrared emission saturation images from 800 nm emission at different depth in liver tissue. Reprinted from Chen et al. (2018) with permission from Springer Nature. **(B)** Left: super-resolution images of the 4% Tm-doped Upconversion nanoparticles (UCNPs) (Liu et al., 2020). Right: comparison of Wiener deconvolution and upconversion nonlinear SIM (U-LSIM) image of the a 51.5 μm liver tissue slice. Scale bar: 1 μm. Reprinted from Liu et al. (2020) with permission from American Chemical Society. **(C)** An enlarged bright view and dark field of glass catfish stained by fluorescent organic nanoparticles (FONPs), and the corresponding confocal and STED images (Xu Y. et al., 2020). Reprinted from Xu Y. et al. (2020) with permission from American Chemical Society. **(D)** SIM imaging of C. elegans labeled with Lyso-Tracker Red (LTR) and developed small molecule dye HD-Br (Fang et al., 2019). Reprinted from Fang et al., 2019 with permission from American Chemical Society. **(E)** Image showing a live mice in supine position after tail injection of C18-PMH-PEG-Ag2Se QDs (Dong et al., 2013). Reprinted from Dong et al. (2013) with permission from American Chemical Society. **(F)** Representative NIR-II dyes used for *in vivo* imaging (Lei and Zhang, 2020). Reprinted from Lei and Zhang (2020) with permission from John Wiley and Sons.

Newly emerging field of *in vivo* supper resolution imaging predicts a significant and large demand for fluorophores in the NIR windows. Current NIR fluorophores are mostly developed based on small organic dyes, inorganic materials, or organic-inorganic hybrid materials. For example, the lanthanide-doped upconversion nanoparticles (UCNPs) have been introduced in super-resolution imaging because of the properties of low excitation power, narrow-band emission, zero auto-fluorescence, large anti-Stokes shifts and high-photostability (Dong et al., 2020; Hu et al., 2020). In 2018, Jin’s group developed near-infrared emission saturation (NIRES) nanoscopy applying UCNPs to realize super-resolution imaging in deep tissue. By employing a doughnut beam excitation from a 980 nm diode laser and detecting at 800 nm, they achieved a resolution of sub 50 nm through 93 μm thick liver tissue (Figure 7A) (Chen et al., 2018). Following this fascinating work, several NIR-based fluorophores developed for *in vivo* SRM were reported. In 2020, Jin and co-workers developed an upconversion nonlinear SIM (U-NSIM) techniques for fast super-resolution imaging through thick tissues using ytterbium (Yb^3+^) and thulium (Tm^3+^) codoped UCNPs as the imaging probe (Liu et al., 2020). They obtained super-resolved image with a resolution below 131 nm and an imaging rate of 1 Hz using this probe (Figure 7B). In 2019, Meng’s group utilized deep-red fluorescent organic nanoparticles (FONPs) DBTBT-4C8 to *in vivo* imaging of transparent glass catfish by STED nanoscopy (Figure 7C) (Xu Y. et al., 2020). In addition, Diao et al. synthesized a near-infrared lysosome-targeted imaging probe HD-Br and applied the probe for 3D lysosomes imaging in live *Caenorhabditis elegans* (Figure 7D) (Fang et al., 2019).

Furthermore, longer wavelength of light results in higher photon penetration depth, which will achieve better performance for *in vivo* application. The pioneering bioimaging using NIR-II fluorophore was reported in 2009. Dai’s group presented tumor vessels beneath thick skin with high-resolution by using the single-walled carbon nanotubes (SWNTs) as an imaging agent (Welsher et al., 2009). In 2012, they obtained high spatial resolution of about 30 µm and temporal resolution of no more than 200 ms/frame for small vessel imaging at 1∼3 mm deep in the tissue with the SWNTs probe (Hong et al., 2012). Besides the SWNTs, the NIR-II quantum dots (QDs) were also reported for *in vivo* imaging (Chen G. et al., 2014). Wang’s group developed a Ag_2_Se QDs with emission centered at 1,300 nm, which have been successfully employed in organs and blood vessels imaging with a high signal-to-noise ratio (Figure 7E) (Dong et al., 2013). In 2015, an NIR-II small-molecule dye CH1055 was used to observe brain tumors in mice at a depth of ∼4 mm (Antaris et al., 2016). Furthermore, Zhang et al. reviewed recent developed NIR-II fluorescent dyes and listed major classes of the probes in Figure 7F (Lei and Zhang, 2020). Although these NIR-II probes have a deep imaging depth, when using in conventional microscopy, the resolution was limited to micrometer scale. Therefore, there are many opportunities for these probes to be used in *in vivo* super resolution imaging.

## Applications of *In Vivo* SRMs in Biological Systems

### Neurosciences

Highly efficient working of the brain mirrors its multiscale complex organization. Altered neuronal morphology and abnormal dynamic actives impair cellular functions, as is observed in various kinds of neurodegenerative disorders such as Parkinson’s and Alzheimer’s diseases (Badawi and Nishimune, 2020; Minehart and Speer, 2020; Walker and Herskowitz, 2020). Hence, visualizing detailed neuronal morphology could promote understanding of the mechanisms underlying neuronal activities and plasticity as well as the diseased-associated disorders. In particular, neurons are not static but changing during brain activities. To analyse their structure and function physiologically, it is essential to study them in living animals (Carrier et al., 2020; Okabe, 2020). Indeed, neuroscience is one of the first disciplines to which *in vivo* SRM was applied.

**FIGURE 8:**
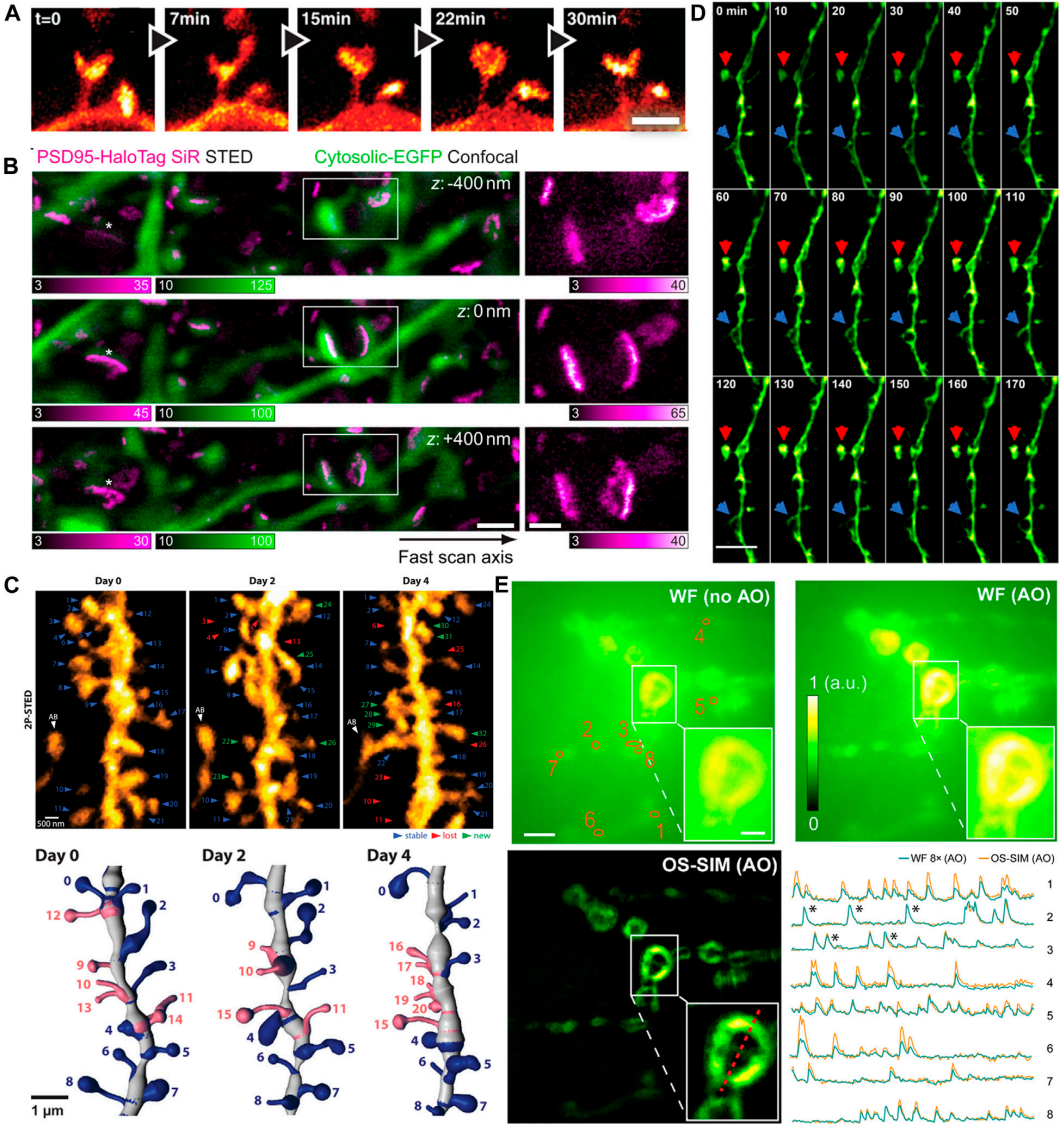
Examples of *in vivo* super-resolution images in the field of neurosciences. **(A)** Dynamics of spines in the molecular layer of the somatosensory cortex with STED microscopy (Berning et al., 2012). Scale bar, 1 μm. Reprinted from Berning et al. (2012) with permission from American Association for the Advancement of Science. **(B)** The 3D images of PSD95 scaffold morphologies in living mouse. Scale bars, 1 μm in original images, 500 nm in magnified views (Masch et al., 2018). Reprinted from Masch et al. (2018) with permission from National Academy of Sciences of the United States of America. **(C)** 2P-STED images of the dendrite over 4 days (Pfeiffer et al., 2018). Reprinted from Pfeiffer et al. (2018) with permission from eLife Sciences. **(D)** Time-lapse *in vivo* SIM images showing structural dynamics of a dendrite in the brain of a Thy1-GFP line M mouse after KCl injection. Arrows point to highly dynamic structures (Turcotte et al., 2019). Scale bar: 4 µm. Reprinted from Turcotte et al. (2019) with permission from National Academy of Sciences of the United States of America. **(E)** *In vivo* functional imaging of quantal releases of a *Drosophila* larva neuromuscular junction with AO OS-SIM (Li et al., 2020). Calcium transients from eight orange regions in wild field view. Scale bars indicated 5 µm in original images and 2 µm in the insets. Reprinted from Li et al. (2020) with permission from American Association for the Advancement of Science.

The first *in vivo* super resolution imaging was carried out with STED microscopy in the somatosensory cortex of an anaesthetized mouse by Stefan W. Hell’s group. They found the morphological changes of the dendritic spines at the head and neck regions in the adult animal brain (Figure 8A) (Berning et al., 2012). Following this work, they observed the actin rearrangement in dendritic spines in a mammalian brain *in vivo*. Subsequently, they provided high-quality super resolution images of the key scaffolding protein postsynaptic density 95 (PSD95) at the postsynaptic membrane by a combination of STED microscopy and endogenous protein labeling method. They showed the PSD95 scaffolds appeared to be continuous structures *in vivo* (Masch et al., 2018), not a fragmented or clustered distribution revealed in previous studies using dissociated rat hippocampal or cortical neurons *in vitro* (Figure 8B) (Hruska et al., 2018; MacGillavry et al., 2013). In 2018, by using 2P-STED microscopy, U Valentin Nagerl et al. showed a twice higher spine density than what was reported in previous works by traditional 2P microscopy, and they disclosed that about 40% of spines turned over within 4 days (Figure 8C) (Pfeiffer et al., 2018).

In recent years, advanced SRMs have not only provided sophisticated optical images of neuronal structures but also revealed the rapid dynamic behaviors in neurosciences. Owing to the relative low illumination intensity and fast imaging acquisition, SIM has been widely implemented to monitor various dynamic events at high frame rates. As mentioned above, Na Ji’s group developed a series modified SIM systems to realize super resolution imaging *in vivo*. They utilized the OA-SIM technique to visualize the fine structural dynamics across 170 min (Figure 8D) (Turcotte et al., 2019). In 2020, they demonstrated a robust quantal synaptic imaging using the *Drosophila* larval neuromuscular junction as a model by genetically encoded calcium indicator (GECI) GCaMP6 (Li et al., 2020). The calcium activity of these terminals was recorded at 25-Hz OS-SIM frame rate (Figure 8E). Although resolution of several tens of nanometers is now obtainable *in vivo*, the applications of SRM to map neuronal morphology and connectivity are still under way.

### Pathology

The conventional morphology-based pathology relies on identifying structural abnormal changes on stained cells and tissues (Si et al., 2020). Clinical imaging techniques such as magnetic resonance and ultrasound imaging enable macroscale detection of the position and morphology of tissues and their obvious changes over time, nevertheless lack cellular- or molecular-scale description of the lesions (Liu and Xu, 2019). At present, optical imaging has been an indispensable tool for preclinical evaluation of diseases. It allows pathologists and biomedical researchers to view 3D volumetric tissue architecture at high resolution and to study pathobiology in both breadth and depth.

**FIGURE 9:**
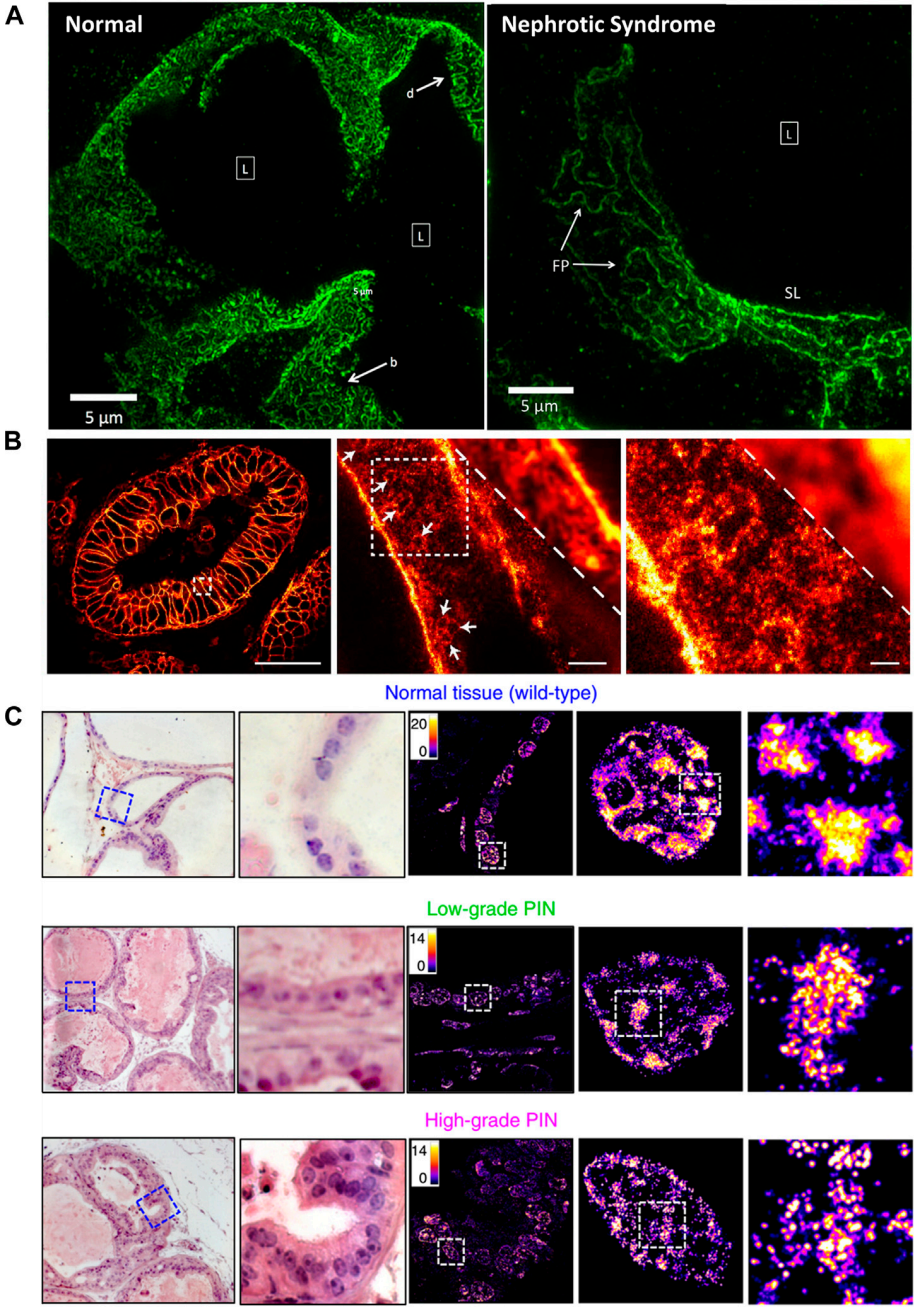
Applications of SRMs in pathobiology. **(A)** Comparison of podocin-stained renal biopsies form normal and nephrotic disease tissue slice by SIM technology (Pullman et al., 2016). Scale bar: 5 µm. Reprinted from Pullman et al. (2016) with permission from the Optical Society of America. **(B)** The diffraction-limited image and STED super-resolution image on sectioned stored tissue of human rectal cancer (Ilgen et al., 2014). Scale bars: 50 µm in original image, 2 µm in magnified images. Reprinted from Ilgen et al. (2014) with permission from Public Library of Science. **(C)** Visualization of disrupted heterochromatin structures in prostate neoplasia by PathSTORM (Xu J. et al., 2020). Reprinted from Xu et al. (2020) with permission from Springer Nature.

In many clinical cases, tissue taken from patients during surgery is stored by formalin- or paraffin-fixed method for disease diagnosis or decision on postoperative treatment plans and subsequent studies. SRMs are introduced to investigate the morphological changes and molecular distribution of some sub-diffraction structures in fixed pathological tissue. Kishan Dholakia et al. performed super-resolution imaging on frozen sections of renal biopsies for podocin protein (Figure 9A) (Pullman et al., 2016). The field of view was approximately 50 times larger than Transmission Electron Microscope (TEM) images. They found that podocin distribution in nephrotic disease biopsies were greatly different from that in normal section. As shown in Figure 9B, the STED microscopy was introduced to investigate detailed distribution of the human epidermal growth factor receptor 2 (HER2) on archived clinical paraffin-embedded rectal cancer tissues (Ilgen et al., 2014). Recently, in 2020, Liu and coworkers employed STORM technique for studying pathological tissue, which named as PathSTORM. They discovered a gradual fragmentation of higher-order chromatin assembling during the all stages of carcinogenesis (Figure 9C) (Xu J. et al., 2020). Although SRM makes a remarkable impaction on the largely unexplored region of molecular structure in diseases assessment, there are still many challenges for the application of SRMs in future clinical diagnosis, such as the sample preparation and tissue labeling.

### Microvessel

The morphological assessment of microvascular network in its natural environment provides a unique perspective for understanding the occurrence and development of infection, hypertension, diabetes, ischemia, cancer and other diseases (Si et al., 2020). As have discussed above, the ultrasound super resolution microscopy (USRM) has been developed for imaging the vasculature *in vivo* beyond the acoustic diffraction limit by the aid of microbubbles (Yoon et al., 2018). This technique can not only obtain the microvasculature in deep tissue, but also provide an accurate blood velocity map.

**FIGURE 10:**
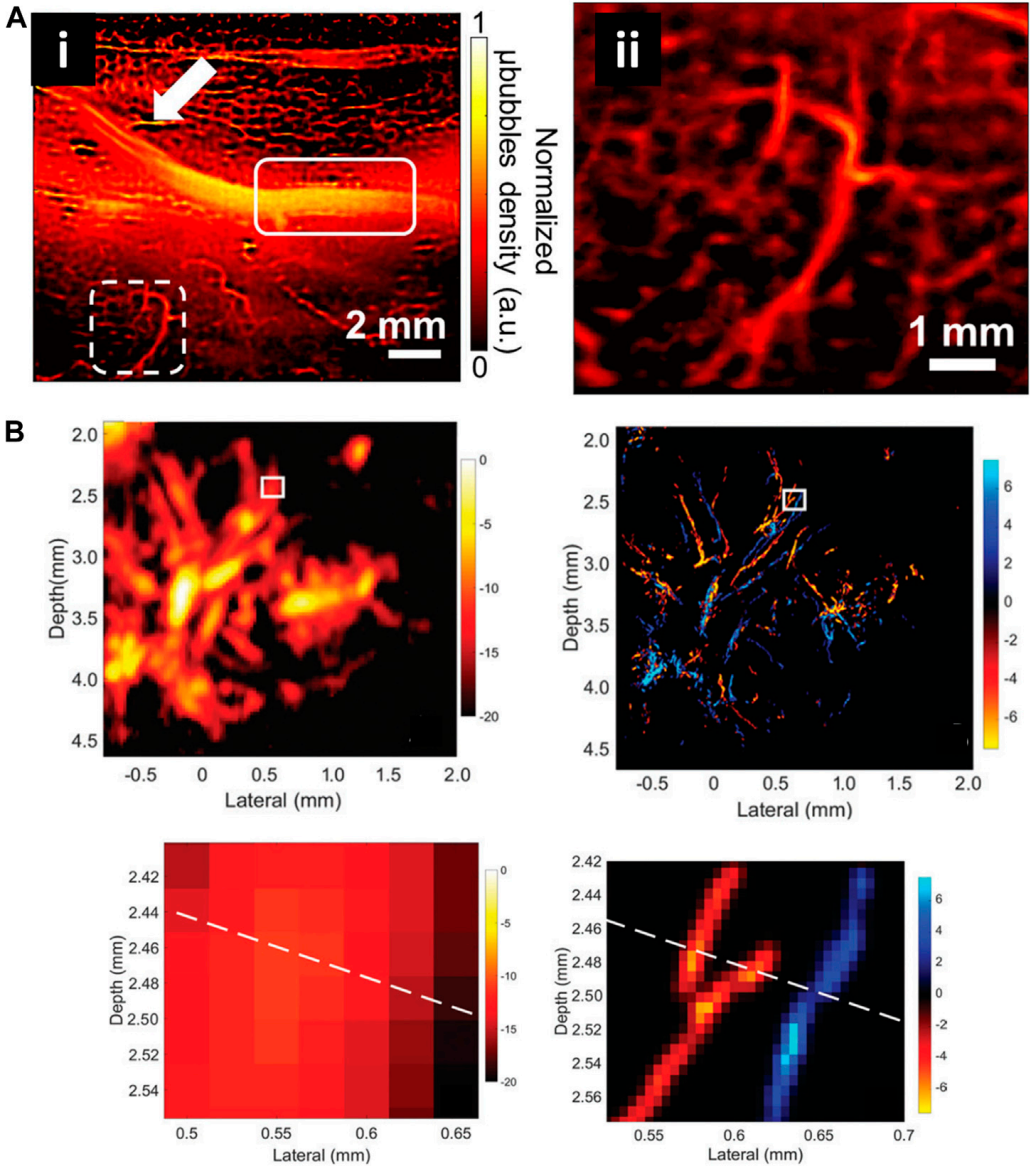
Super-resolved vessel maps by ultrasound localization microscopy. **(A)** The super-resolution ultrasound image of vascular based on Verasonics system (i) and enlarged view of white dashed rectangle (ii) (Yu et al., 2018). Reprinted from Yu et al. (2018) with permission from Springer Nature. **(B)** Acoustic subaperture processing (ASAP)-enhanced power doppler image and ultrasound super-resolution image (Zhu et al., 2019). Magnified views showed the details of boxed region. Reprinted from Zhu et al. (2019) Radiological Society of North America.

Currently, several USRM technologies have been successfully applied *in vivo* for imaging microvessels in different organs. Robert J. Eckersley et al. in 2014 realized super resolution imaging of blood velocity by tracking individual microbubbles at depth of more than 1 cm (Christensen-Jeffries et al., 2015). They detected microvasculature of mouse ear with a resolution of 20 µm. Subsequently, Olivier Couture et al. successfully reconstructed the rat brain vasculature network with FWHM as small as 9 μm using a 20 MHz linear array transducer (Errico et al., 2015). Kang Kim et al. enhanced the temporal resolution for imaging vessels over cardiac motion vasa vasorum in the rabbit atherosclerosis model (Figure 10A) (Yu et al., 2018). Meng-Xing Tang et al. developed 3D ultrasound super resolution technique to visualize rabbit lymph node microvascular structures and blood flow dynamics with a resolution of 30 μm, which achieve a 15-fold improvement over existing doppler techniques (Figure 10B) (Zhu et al., 2019). More recently, in 2020, the micron-level choroidal and retrobulbar vessels around the optic nerve head were successfully reconstructed in rabbit eye by using ultrasound microbubble localization approach (Qian et al., 2020). With the development of USRM techniques, we believed that this method could be of great value for observing pathological changes or therapeutic effect on the microvasculature *in vivo*.

## Conclusions and Future Challenges

Super-resolution imaging techniques have pushed a vital step forward in how researchers view and study for biological questions, significantly expanding our knowledge of molecular interactions and dynamic processes within cells and tissues. However, more complete understanding of how the biomolecules assemble to create animate lives requires observing the cell in its native state. *In vivo* imaging entails huger challenges of sufficient image resolution and imaging depth. Fortunately, as we indicated in this review, more and better SRM techniques become available for *in vivo* imaging. We have outlined a detailed description of currently improved super-resolution microscopes for *in vivo* imaging, including modified STED, SIM, RESOLFT, LLSM, and ULM and listed the biological applications that benefit from these techniques. Among these advanced techniques, STED method is best suited and widely applied for *in vivo* imaging because of the high resolution, simple data processing, and intrinsic 3D sectioning capability, which is ideal for tissue imaging in living species. Additionally, SIM also gained a lot of interest in *in vivo* applications as minimal amount of photo-damage and commonly used labelling methods. In comparison, the applicability of RESOLFT, LLSM, and ULM in *in vivo* imaging is restricted mainly due to the necessaries for more professional technical modification as well as more rigorous operation.

As we look toward the future of the field, it is clear that there are some key challenges still need to be resolved in the future development of *in vivo* SRM. We would like to conclude this review with some speculative and directional thoughts on the improvements of *in vivo* SRM. First, although the abilities of SRM have expanded in recent years, new strategies and techniques that increase the imaging depth while maintaining a high resolution, especially for 3D imaging, are still required to obtain true information from thicker tissues and whole organs *in vivo*. Correlating diverse imaging methods offer an effective way to realize deep imaging *in vivo*, such as implementing the multiphoton excitation and lattice light sheet illumination pattern for SRMs to enhance imaging depth (Li et al., 2015; Huang et al., 2021), and combining AO with various SRMs to correct sample-induced aberrations (Booth, 2014; Zheng et al., 2017; Johnstone et al., 2019). Second, the labeling probes possessing suitable spectroscopic and chemical properties has become a major bottleneck to unleash the full potential of SRMs. To create super-resolution maps of the local environment, both biologists and chemists could be stimulated to develop new labeling strategies that not only have far red emitting but also minimally disturb the tagged biomolecule (Fernandez-Suarez and Ting, 2008; Wang et al., 2018; Reja et al., 2021). Third, improvements in real-time imaging combining with faster temporal resolution are necessary to resolve time dependent processes *in vivo*. sCMOS cameras have been used as an alternative to the EMCCDs in some super-resolution imaging cases, especially for the dynamic tracking (Schermelleh et al., 2019). With increasing enhancement on the sensitivity and quantum yield of these detectors, faster frame rates and larger pixel arrays will become generally accessible. Last but not the least, the animal preparation is also a vital step for *in vivo* imaging. Any movement caused by vibrations of the microscopy, unavoidable essential functions of the living species including heartbeat, pressure pulse, or thermal drift, will disturb the imaging process (Steffens et al., 2012; Steffens et al., 2020). Advanced imaging strategies employed on the awake or anesthetic animal such as the cranial window technique and optical alignment device should keep pace with the SRM techniques.

We believe that the development of SRMs from *vitro* to *vivo* is in its infancy, and in the coming years, researchers will see continued advances across diverse techniques, enabling new and multifarious applications to answer more valuable biological questions.
