# Corrigendum: Super-Resolution Microscopy: Shedding New Light on *In Vivo* Imaging

**DOI:** 10.3389/fchem.2021.795767

**Published:** 2021-11-22

**Authors:** Yingying Jing, Chenshuang Zhang, Bin Yu, Danying Lin, Junle Qu

**Affiliations:** Key Laboratory of Optoelectronic Devices and Systems of Ministry of Education and Guangdong Province, College of Physics and Optoelectronic Engineering, Shenzhen University, Shenzhen, China

**Keywords:** super-resolution techniques, *in vivo* imaging, labeling strategies, near-infrared fluorescent probes, *in vivo* applications

In the original article, there was a mistake in the legend for **Figure 9A** as published. The correct legend appears below.

**(A)** Comparison of podocin-stained renal biopsies form normal and nephrotic disease tissue slice by SIM technology (**Pullman et al., 2016**). Scale bar: 5 µm. Reprinted from **Pullman et al. (2016)** with permission from the Optical Society of America.

In the original article, the reference for **Figure 9A** was incorrectly written as:

Unnersjö-Jess, D., Scott, L., Blom, H., and Brismar, H. (2016). Superresolution Stimulated Emission Depletion Imaging of Slit Diaphragm Proteins in Optically Cleared Kidney Tissue. *Kidney Int.* 89, 243–247. doi: 10.1038/ki.2015.308.

It should be:

Pullman J. M., Nylk J., Campbell E. C., Gunn-Moore F. J., Prystowsky M. B., and Dholakia K. (2016) Visualization of Podocyte Substructure with Structured Illumination Microscopy (SIM): a New Approach to Nephrotic Disease. *Biomed Opt. Express*, 7, 302–311. doi: 10.1364/BOE.7.000302.

In the original article, there was an error. **Figure 3** does not contain part E. The text in **Labeling approaches, Genetically Encoded Probes**, Paragraph 2 was changed to read: “For example, several experiments for in vivo SRM applied the heterozygous TgN (Thy1-EYFP) mice expressing enhanced yellow fluorescent protein (EYFP) or GFP, EGFP, which is under the control of the regulatory element from the thy1 gene (**Figure 1A**) (**Berning et al., 2012; Bethge et al., 2013**)”.

The authors apologize for this error and state that this does not change the scientific conclusions of the article in any way. The original article has been updated.

